# Associations of Common Variants at *APLN* and Hypertension in Chinese Subjects with and without Diabetes

**DOI:** 10.1155/2012/917496

**Published:** 2012-12-17

**Authors:** Rong Zhang, Jingyi Lu, Cheng Hu, Congrong Wang, Weihui Yu, Feng Jiang, Shanshan Tang, Yuqian Bao, Kunsan Xiang, Weiping Jia

**Affiliations:** Department of Endocrinology and Metabolism, Shanghai Jiao Tong University Affiliated Sixth People's Hospital, Shanghai Diabetes Institute, Shanghai Key Laboratory of Diabetes Mellitus, Shanghai Clinical Center for Diabetes, 600 Yishan Road Shanghai, 200233, China

## Abstract

*Background*. Apelin, the endogenous ligand for the APJ receptor, has a potent hypotensive effect via a nitric oxide-dependent mechanism in vivo. The aim of the study was to investigate the association between the common variants of apelin gene (*APLN*) and hypertension, which was reported recently in a Chinese Han population with and without diabetes. *Methods*. Three single nucleotide polymorphisms (SNPs) on *APLN* were genotyped in 3156 diabetic patients and 3736 nondiabetic individuals. For non-diabetic subjects, 1779 were enrolled in stage 1 and 1757 were recruited for validation. A meta-analysis combining the two stages was carried out to obtain the overall effect. *Results*. In diabetic patients, no significant associations of the three SNPs with hypertension were observed. In contrast, we found that rs2235306 was associated with hypertension in non-diabetic males after adjusting for covariates (OR = 1.19, *P* = 0.039) while rs2235307 and rs3115759 displayed no evidence of association in both genders. One haplotype, C-C-A, also showed an association with hypertension (OR = 1.47, *P* = 0.032) only in men. However, analysis in stage 2 and meta-analysis did not support these findings. *Conclusions*. We conclude that common variants on *APLN* are not associated with the prevalence of hypertension in the Chinese.

## 1. Introduction

Essential hypertension is a major risk factor for many common causes of morbidity and mortality including stroke, myocardial infarction, heart failure, and end-stage renal disease [1–3]. Approximately, 30–50% of blood pressure variation in the general population is determined by genetic factors [4, 5] and a variety of gene variants have been shown to be associated with essential hypertension [6, 7].

Apelin, the endogenous ligand for the APJ receptor (an orphan G-protein-coupled receptor), is a bioactive peptide that is expressed in a wide variety of tissues [8–11]. The apelin gene (*APLN*) in humans is located on chromosome Xq25–26.1, which encodes a 77-amino acid prepropeptide that is cleaved into isoforms of varying lengths [8–13]. During the recent years, there is a mounting evidence that apelin has pleiotropic effects on lipid and glucose metabolisms [14–16] and therefore is correlated with metabolic disorders including diabetes. On the other side, since the apelin/APJ system has high sequence homology to the angiotensin II/angiotensin receptor (AII/AT) system [17], it has gained much research interest regarding its role in blood pressure control. By binding to APJ, apelin exerts a potent hypotensive effect via a nitric oxide-dependent mechanism in vivo [12, 18, 19]. In accord with this, circulating apelin was reported to be decreased in patients with essential hypertension [20, 21], suggesting an important role for the apelin/APJ system in human blood pressure regulation.

To date, two studies have reported the associations of common genetic variants of *APLN* with hypertension [22, 23]. However, genetic findings need replication. Thus, we sought to investigate the relationship between *APLN* tagging SNPs and hypertension in a community-based population free of diabetes, excluding the confounding effect of diabetes on hypertension. On the other hand, hypertension and type 2 diabetes frequently coexist. There is a compelling evidence that hypertension could significantly increase the risk of cardiovascular diseases in diabetic patients [24], indicating an urgent need for identifying hypertension susceptible genes in this subset of individuals [25]. Moreover, dysfunctional endothelium-dependent vasodilation and impaired nitric oxide bioavailability have been reported in diabetes [26–28], raising the possibility that apelin may interact with diabetes in the regulation of blood pressure. In this context, another aim of the current study was to examine the effect of *APLN* genetic variants on hypertension in patients with diabetes.

## 2. Materials and Methods

### 2.1. Subjects

Diabetic patients were selected from the inpatient database of the Shanghai Diabetes Institute. Diabetes was defined according to the 1999 WHO criteria (fasting plasma glucose ≥7.0 mmol/L and/or 2 h plasma glucose ≥11.1 mmol/L). Nondiabetic subjects were enrolled from the Shanghai Diabetes Study [29], a community-based epidemiological survey for diabetes. Briefly, the Huayang and Caoyang communities, two middle-income communities in Shanghai, were selected for the survey. The target population included unrelated residents over 40 years of age who had been living in Shanghai for more than 10 years. Normal glucose regulation was confirmed by fasting plasma glucose (<6.1 mmol/L) and 2 h plasma glucose after oral glucose tolerance test (<7.8 mmol/L). All subjects were of the Han Chinese ancestry and resided in Shanghai or nearby regions. Individuals who suffered from secondary hypertension, cancer, severe disability or severe psychiatric disturbance were excluded. In the analyses restricted to non-diabetic individuals, we used a two-stage strategy. In stage 1, a total of 1779 (728 men and 1051 women) subjects were recruited. In stage 2, we additionally enrolled 1757 (668 men and 1089 women) subjects to validate the findings from stage 1, following the same inclusion and exclusion criteria as in stage 1. As for diabetic patients, 3156 were finally recruited. 

General anthropometric parameters, including height, weight, and blood pressure, were measured in all participants. BMI was calculated as weight (kg)/height^2^ (m)^2^. Blood pressure was measured twice with standard mercury sphygmomanometers in each participant while seated, after 5 minutes of rest and the average of two readings was taken. Essential hypertension was defined as blood pressure ≥140/90 mm Hg and/or the current use of antihypertensive medications. While the information on smoking was not available in stage 1, we obtained this information using questionnaires in stage 2. The study protocol was approved by the Institutional Review Board of Shanghai Jiao Tong University Affiliated Sixth People's Hospital, Shanghai, China. All participants gave informed consent prior to the study. 

### 2.2. Clinical Laboratory Tests

Blood samples were collected after an overnight fast. Serum lipid profiles, including total cholesterol, triglyceride, high-density lipoprotein cholesterol (HDL-C), and low-density lipoprotein cholesterol (LDL-C), were measured with a type 7600-020 automated analyzer (Hitachi, Tokyo, Japan). 

### 2.3. SNP Selection and Genotyping

Genomic DNA was extracted from peripheral blood leukocytes using the standard phenol-chloroform method. Based on the database of the HapMap Chinese Hans in Beijing (CHB) Group, three SNPs (rs2235307, rs2235306, and rs3115759) that captured all variants with minor allele frequencies over 0.05 in the *APLN *region under the threshold of *r* ^2^ = 0.8 were selected in this study by Tagger incorporated in Haploview (version 4.1) [30]. rs2235307, rs2235306, and rs3115759 map to intron 1, intron 2, and exon 3 of *APLN*, respectively. The genotyping was performed by matrix-assisted laser desorption ionization time of flight mass spectroscopy (MassARRAY Compact Analyzer, Sequenom, San Diego, CA, USA). The genotyping success rates for rs2235307, rs2235306, and rs3115759 were 98.7%, 98.7%, and 99.5%, respectively. The average concordance rate based on 100 duplicate pairs was 99.3% for each SNP.

### 2.4. Statistical Analysis

As *APLN* is located on the X chromosome, the genotype-hypertension analysis was conducted in men and women separately. Allele, genotype, and haplotype frequencies for cases and controls were compared by using *χ* ^2^-test. Logistic regression analysis after adjustment for age, BMI, and FPG (for non-diabetic subjects) or diabetes duration (for diabetic patients) was carried out to evaluate the effect of genotypes and alleles on the prevalence of hypertension. Odds ratios (ORs) with 95% confidence interval (95% CI) were presented. A pairwise linkage disequilibrium (LD) was estimated by calculating *|D*′*|* and *r* ^2^ using Haploview (version 4.1). The haplotype block structure was determined using confidence interval algorithm [31], and haplotype frequencies were estimated by the expectation-Maximization algorithm [32] using Haploview (version 4.1). Haplotypes with a frequency <0.05 were excluded. All statistical analyses were performed by SAS (version 8.0; SAS Institute Inc., Cary, NC, USA) unless specified otherwise. A two-tailed *P*  value < 0.05 was considered statistically significant. Combined ORs and 95% CIs were calculated with Review Manager (version 5.0). Interstudy heterogeneity was estimated using the *χ* ^2^-based Q statistic. Heterogeneity was considered statistically significant when *P* < 0.05. *I* ^2^ was also tested, which describes the percentage of total variation in point estimates attributable to genuine variation rather than sampling error. If heterogeneity existed, data were analyzed using a random effect model while in the absence of heterogeneity, a fixed effect model was used.

## 3. Results

Table 1 presents the characteristics of diabetic patients. The characteristics of non-diabetic subjects are shown in Supplemental Table 1 (See Supplementry Material available at doi:10.1155/2012/917496). All the three SNPs conformed to the Hardy-Weinberg equilibrium in both controls and total participants.

For diabetic patients, the allele and genotype distributions of the SNPs genotyped did not differ significantly between hypertensive and normotensive individuals in males (Table 2) and females (Table 3), respectively. Adjusting for covariates including age, BMI, and diabetes the duration, the logistic regression analysis did not reveal any significant associations (Tables 2 and 3), either. 

**Figure 1:**
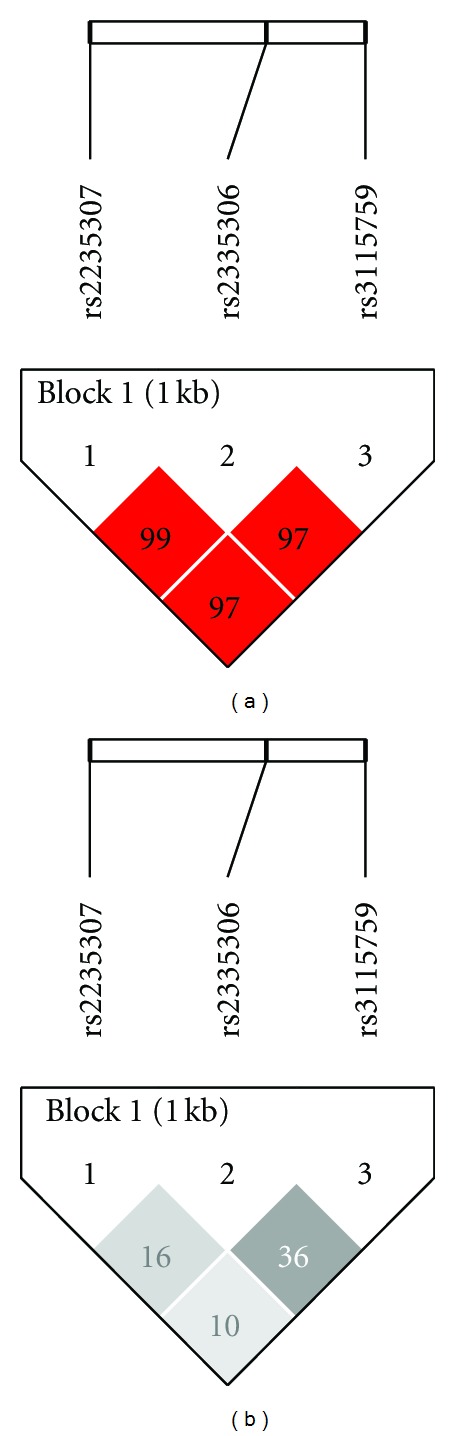
Pairwise linkage disequilibriums of SNPs genotyped in the *APLN *region. (a) Numbers represent *|D*′*|* expressed as a percentage. (b) Numbers represent *r* ^2^ expressed as a percentage.

Based on the *|D*′*|* and *r* ^2^ values, the three SNPs were located in one block (Figure 1). Four haplotypes, constructed by rs2235307, rs2235306, and rs3115759, with a frequency of >0.05 were observed in our study samples (Table 4). None of them exhibited a significantly differed distribution between hypertensive and normotensive participants in the diabetes group.

Concerning non-diabetic participants (stage 1), we found that rs2235306, but not rs2235307 and rs3115759, was associated with the prevalence of hypertension only in men (Supplemental Tables 2 and 3) after adjusting for covariates (OR = 1.19, 95% CI: 1.01–1.40, *P* = 0.039), with the minor allele (C) being more frequent in hypertensive subjects. Moreover, the C-C-A haplotype (rs2235307-rs2235306-rs3115759) was related to with a higher risk of hypertension (OR = 1.47, 95% CI 1.09–2.00, *P* = 0.032) in men (Supplemental Table 4). No association of the other three haplotypes with hypertension was observed.

Since the rs2235306 C allele and the associated haplotype C-C-A correlated with a higher hypertension risk in non-diabetic males, we genotyped the three SNPs in an additional group (stage 2, *n* = 1757) of non-diabetic subjects. In these samples, no evidence for the associations of rs2235306 and the C-C-A haplotype with hypertension was observed (Supplemental Tables 2, 3, and 4). Next, we combined the two stages with meta-analysis (Supplemental Table 5). None of the three SNPs and the four haplotypes was significantly associated with hypertension.

## 4. Discussion

The current study genotyped three tagging SNPs of *APLN* region in a Chinese population with and without diabetes. The pilot data showed that the minor allele C of rs2335306 and the C-C-A haplotype (rs223537-rs2235306-rs3115759) were associated with a higher risk of hypertension in men with normal glucose regulation. However, our second stage samples failed to replicate this finding, and the meta-analysis also exhibited a negative result. 

In 1998, Tatemoto et al. [13] isolated a 36-amino acid peptide from bovine stomach extracts and named it apelin, the endogenous ligand to an orphan G-protein-coupled receptor APJ. In human beings, preproapelin mRNA is abundant in the central nervous system, placenta and in more moderate concentrations in the kidney, heart, lungs, mammary gland, and adipocytes. Since its discovery, many of the initial investigations of the apelin/APJ system, as it relates to the cardiovascular system centered on its role in blood pressure control [12, 18, 19]. In male Wistar rats, the administration of aplein could lower systolic and diastolic blood pressure, which persisted for several minutes [19, 33]. Besides, a nitric oxide-dependent arterial vasodilation effect of apelin was also observed in vivo in men [18].

It is notable that no study has investigated whether there is difference between males and females regarding the effect of apelin on blood pressure. However, Newson et al. [34] reported that the apelin receptor (APJ) had a gender-specific function in peripheral immune activation of the hypothalamic-pituitary-adrenal axis, suggesting that the possible gender-specific influence of apelin/APJ on blood pressure may be an issue to address in the future.

Recently, Li and his colleagues [22] have assessed the associations of polymorphisms within *APLN* with essential hypertension in a family-based study and reported a positive result. This association was next validated in a general population by Niu et al. [23]. Nonetheless, it is notable that the associated SNPs in these two studies were not consistent. For instance, T-1860C, which was found to affect the risk of hypertension in the former study, was not successfully replicated in the latter one, where rs3761581 was observed to be risk conferring. Moreover, the *P* values for the associations were not solid enough (*P* = 0.018 and *P* = 0.0156, resp.). Together with our data, it is likely that common variants of *APLN* alone may not exert a pronounced genetic effect on blood pressure. However, the possibility of association could not be fully excluded, as our study might have insufficient power when detecting mild genetic effects of SNPs with low minor allele frequencies. For instance, the diabetic group had less than 50% power to detect an OR of 1.2 for rs2335307 (Supplemental Table 6). On the other hand, it is possible that the interaction between *APLN* and *AGTRL1*, which encodes the receptor of apelin, may have a greater genetic effect on the regulation of blood pressure. Indeed, in the study by Niu et al. [23], the association with hypertension was much more robust in the haplotype analysis, which took variants of both *APLN* and *AGTRL1* into consideration, than in the single-locus study restricted to *APLN*. 

The strength of the current study resides in that a total of 6693 subjects (3156 diabetic patients and 3536 non-diabetic subjects) were recruited, which we believe is the largest sample size of study to evaluate the association between *APLN* and hypertension to date. But there are several limitations that should be mentioned. First, we did not obtain the information on pharmacological treatments, which might have certain effect on blood pressure. Second, common variants of *AGTRL1* were not genotyped. Therefore, the effect of interaction between *AGTRL1* and *APLN* on hypertension remained unknown. Third, we did not measure the circulating apelin concentration in our sample, precluding the analysis for the association between *APLN *genetic variants and circulating apelin levels. 

In summary, we did not find any evidence for the association of common variants on *APLN* with hypertension in either diabetic or non-diabetic subjects. Although a minor effect could not be excluded, our data suggest that common variants of *APLN* may not play a major role in the regulation of blood pressure. Further studies in different populations are needed to confirm this finding. 

## Supplementary Material

The supplemental tables include the characteristics of the non-diabetic subjects, the individual-SNP and haplotype analysis in non-diabetic males and females, the meta-analysis and the power calculation of the current study.

## Figures and Tables

**Table 1:** The characteristics of diabetic patients.

Parameters	Men			Women		
	NT (*n* = 653)	HT (*n* = 997)	*P *	NT (*n* = 457)	HT (*n* = 1049)	*P*
Age (years)	53.92 ± 12.84	61.62 ± 12.30	<0.001	58.8 ± 12.05	65.24 ± 10.43	<0.001
BMI	23.30 ± 3.37	24.62 ± 3.20	<0.001	23.04 ± 3.34	24.60 ± 3.70	<0.001
Onset age (years)	48.77 ± 11.58	53.30 ± 11.49	<0.001	52.63 ± 11.12	56.02 ± 11.09	<0.001
Diabetes duration (years)	5.39 ± 5.47	7.37 ± 6.74	<0.001	7.73 ± 6.48	9.15 ± 7.22	0.030
SBP (mm Hg)	119.95 ± 9.67	141.00 ± 16.71	<0.001	120.79 ± 9.82	144.84 ± 17.84	<0.001
DBP (mm Hg)	75.60 ± 6.40	84.99 ± 9.86	<0.001	74.65 ± 6.69	83.11 ± 9.84	<0.001
Total cholesterol (mmol/L)	4.47 ± 1.00	4.70 ± 1.21	<0.001	4.88 ± 1.06	5.01 ± 1.20	0.097
Triglyceride (mmol/L)	1.79 ± 2.04	1.98 ± 2.05	<0.001	1.67 ± 1.41	2.02 ± 1.81	<0.001
HDL (mmol/L)	1.08 ± 0.30	1.10 ± 0.33	0.330	1.30 ± 0.40	1.26 ± 0.73	<0.001
LDL (mmol/L)	2.89 ± 0.91	3.00 ± 0.94	0.016	3.06 ± 0.88	3.12 ± 0.96	0.299

Continuous variables were means ± SD. Categorical variables were numbers with percentages. NT: normotensive subjects. HT: hypertensive subjects. BMI: body mass index. SBP: systolic blood pressure. DBP: diastolic blood pressure. HDL: high-density lipoprotein cholesterol. LDL: low-density lipoprotein cholesterol.

**Table 2:** Analysis for the association of the three SNPs with hypertension in diabetic males.

SNP	Allele	NT (*n* = 653)	HT (*n* = 997)	OR (95% CI)	*P*	P*
rs2235307	C	520 (80.37%)	809 (82.38%)	0.83 (0.59–1.15)	0.305	0.250
	T	127 (12.93%)	173 (17.62%)			

rs2235306	T	377 (58.27%)	538 (54.73%)	1.17 (0.90–1.50)	0.159	0.239
	C	270 (41.73%)	445 (45.27%)			

rs3115759	A	442 (67.69%)	673 (67.64%)	0.94 (0.72–1.22)	0.983	0.639
	G	211 (32.31%)	322 (32.36%)			

NT: normotensive subjects. HT: hypertensive subjects.

*P values were adjusted for age, body mass index and diabetes duration.

**Table 3:** Analysis for the association of the three SNPs with hypertension in diabetic females.

SNP	Genotype	NT	HT	OR (95% CI)	*P *	P*
rs2235307	CC	288 (63.6%)	712 (68.8%)	0.88 (0.54–1.42)	0.143	0.592
	CT	150 (33.1%)	293 (28.3%)			
	TT	15 (3.3%)	30 (2.9%)			

rs2235306	TT	150 (33.5%)	318 (30.7%)	1.19 (0.80–1.76)	0.562	0.390
	TC	217 (48.4%)	525 (50.6%)			
	CC	81 (18.1%)	194 (18.7%)			

rs3115759	AA	207 (45.3%)	464 (44.2%)	0.78 (0.53–1.15)	0.485	0.212
	AG	193 (42.2%)	472 (45.0%)			
	GG	57 (12.5%)	113 (10.8%)			

NT: normotensive subjects. HT: hypertensive subjects.

*P values were adjusted for age, body mass index, and diabetes duration.

**Table 4:** Analysis for the association of haplotypes with hypertension in diabetic patients stratified by gender.

	Men					Women				
Haplotype	Frequency					Frequency				
			OR (95% CI)	*P*	P*			OR (95% CI)	*P*	P*
	HT	NT				HT	NT			
CCA	0.45	0.42	1.16 (0.95–1.41)	0.558	0.162	0.44	0.42	1.06 (0.91–1.24)	0.160	0.460
CTG	0.32	0.32	0.99 (0.81–1.23)	0.278	0.972	0.32	0.33	0.98 (0.84–1.16)	0.464	0.867
TTA	0.18	0.20	0.89 (0.69–1.15)	0.319	0.371	0.17	0.20	0.84 (0.69–1.02)	0.551	0.083
CTA	0.04	0.06	0.69 (0.45–1.07)	0.793	0.079	0.06	0.05	1.33 (0.93–1.89)	0.068	0.111

Haplotypes were constructed in the order of rs2235307, rs2235306, and rs3115759.

NT: normotensive subjects. HT: hypertensive subjects.

*P values were adjusted for age, body mass index and diabetes duration.
