# Supplementary material for: Associations of Common Variants at APLN and Hypertension in Chinese Subjects with and without Diabetes
Source: Exp Diabetes Res. 2012 Dec 17;2012:917496. doi: 10.1155/2012/917496 (PMC3534217; doi:10.1155/2012/917496)
Supplement: Supplementary file 1 — The supplemental tables include the characteristics of the non-diabetic subjects, the individual-SNP and haplotype analysis in non-diabetic males and females, the meta-analysis and the power calculation of the current study. [file 917496.f1.docx]

The supplemental tables include the characteristics of the non-diabetic subjects, the individual-SNP and haplotype analysis in non-diabetic males and females, the meta-analysis and the power calculation of the current study.

**Supplemental Table 1 The characteristics of individuals without diabetes**

| Parameters | Stage 1 | | | | |  | Stage 2 | | | | |
| --- | --- | --- | --- | --- | --- | --- | --- | --- | --- | --- | --- |
|  | Men | |  | Women | |  | Men | |  | Women | |
|  | NT (n=450) | HT (n=273) |  | NT (n=756) | HT (n=291) |  | NT (n=450) | HT (n=218) |  | NT (n=869) | HT (n=219) |
| Age (years) | 59.01±13.05 | 61.27±12^*^ |  | 53.47±10.97 | 61.29±12.14^*^ |  | 42.35±14.43 | 54.82±12.19^*^ |  | 42.4±12.23 | 55.51±9.27^*^ |
| BMI | 22.53±2.94 | 24.71±3.02^*^ |  | 23.16±3.08 | 25.05±3.46^*^ |  | 23.32±3.3 | 24.83±2.96^*^ |  | 23.02±3.1 | 24.81±3.24^*^ |
| SBP (mm Hg) | 117.13±11.46 | 144.62±14.51^*^ |  | 115.42±11.91 | 146.92±16.07^*^ |  | 116.59±10.11 | 143.68±13.64^*^ |  | 113.86±11.19 | 143.85±11.71^*^ |
| DBP (mm Hg) | 74.69±6.11 | 91.35±7.6^*^ |  | 73.72±6.55 | 89.16±9.28^*^ |  | 75.64±6.96 | 90.56±9.15^*^ |  | 73.63±7.21 | 87.8±9.01^*^ |
| FPG (mmol/L) | 4.93±0.52 | 5.02±0.51^*^ |  | 5±0.48 | 5.07±0.48^*^ |  | 5.01±0.53 | 5.11±0.44^*^ |  | 5.08±0.51 | 5.2±0.46^*^ |
| Total cholesterol (mmol/L) | 4.85±1.06 | 4.97±1.06 |  | 5.02±1.06 | 5.39±1.88^*^ |  | 4.34±0.89 | 4.66±0.98^*^ |  | 4.41±0.85 | 5.07±0.98^*^ |
| Triglyceride (mmol/L) | 1.55±0.92 | 2.02±1.77^*^ |  | 1.46±0.77 | 1.84±1.28^*^ |  | 1.57±1.12 | 1.91±1.37^*^ |  | 1.15±0.75 | 1.79±1.14^*^ |
| HDL (mmol/L) | 1.29±0.28 | 1.25±0.27 |  | 1.38±0.28 | 1.37±0.3 |  | 1.19±0.28 | 1.26±0.31^*^ |  | 1.43±0.3 | 1.36±0.3^*^ |
| LDL (mmol/L) | 3.3±0.92 | 3.31±0.9 |  | 3.37±0.94 | 3.54±1^*^ |  | 2.82±0.73 | 2.91±0.84^*^ |  | 2.74±0.71 | 3.21±0.86^*^ |
| Smoking habit |  |  |  |  |  |  |  |  |  |  |  |
| non-smoker | / | / |  | / | / |  | 87 (22.9%) | 29 (16.3%) |  | 517 (97.4%) | 125 (99.2%) |
| former smoker | / | / |  | / | / |  | 21 (5.5%) | 15 (8.4%) |  | 3 (0.6%) | 0 (0%) |
| current smoker | / | / |  | / | / |  | 272 (71.6%) | 134 (75.3%) |  | 11 (2.0%) | 1 (0.8%) |
| *P* value |  | / |  |  | / |  |  | 0.114 |  |  | 0.725 |

Continuous variables were means±SD. Categorical variables were numbers with percentages.

NT, normotensive subjects. HT, hypertensive subjects. BMI, body mass index. SBP, systolic blood pressure. DBP, diastolic blood pressure. HDL, high-density lipoprotein cholesterol. LDL, low-density lipoprotein cholesterol.

^*^*P*<0.05 compared with normotensive subjects.

**Supplemental Table 2 Analysis for the association of the three SNPs with hypertension in men without diabetes**

| SNP | Allele | Stage 1 | | | |  | Stage 2 | | | |
| --- | --- | --- | --- | --- | --- | --- | --- | --- | --- | --- |
|  |  | NT (n=450) | HT (n=278) | OR (95% CI) | *P*^*^ |  | NT (n=450) | HT (n=218) | OR (95% CI) | *P*^*^ |
| rs2235307 | C | 356 (80.2%) | 226 (84.6%) |  |  |  | 378(84.4%) | 180(82.6%) |  |  |
|  | T | 88 (19.8%) | 41 (15.4%) | 0.90 (0.73-1.12) | 0.351 |  | 70(15.6%) | 38(17.4%) | 1.05 （0.82-1.35） | 0.678 |
| rs2235306 | T | 274 (61.4%) | 140 (51.9%) |  |  |  | 237(53.5%) | 120(55.1%) |  |  |
|  | C | 172 (38.6%) | 130 (48.1%) | 1.19 (1.01-1.40) | 0.039 |  | 206(46.5%) | 98(44.9%) | 0.97 （0.81-1.16） | 0.733 |
| rs3115759 | A | 288 (64.3%) | 191 (69.9%) |  |  |  | 303(67.9%) | 144(66.4%) |  |  |
|  | G | 160 (35.7%) | 82 (30.1%) | 0.86 (0.72-1.02) | 0.078 |  | 143(32.1%) | 73(33.6%) | 1.03 （0.85-1.25） | 0.746 |

NT, normotensive subjects. HT, hypertensive subjects.

^*^*P* values were adjusted for age, body mass index and fasting plasma glucose.

**Supplemental Table 3 Analysis for the association of the three SNPs with hypertension in women without diabetes**

| SNP | Genotype | Stage 1 | | | |  | Stage 2 | | | |
| --- | --- | --- | --- | --- | --- | --- | --- | --- | --- | --- |
|  |  | NT (n=756) | HT (n=295) | OR (95% CI) | *P*^*^ |  | NT (n=756) | HT (n=295) | OR (95% CI) | *P*^*^ |
| rs2235307 | CC | 509 (68.1%) | 186 (64.8%) |  |  |  | 579(66.7%) | 133(60.7%) |  |  |
|  | CT | 199 (26.6%) | 93 (32.4%) |  |  |  | 253(29.2%) | 75(34.3%) |  |  |
|  | TT | 40 (5.3%) | 8 (2.8%) | 0.98 (0.75-1.27) | 0.867 |  | 36(4.1%) | 11(5.0%) | 1.35 （1.02-1.80） | 0.038 |
|  |  |  |  |  |  |  |  |  |  |  |
| rs2235306 | TT | 238 (31.9%) | 92 (31.7%) |  |  |  | 265(30.5%) | 69(31.5%) |  |  |
|  | TC | 362 (48.6%) | 144 (49.7%) |  |  |  | 412(47.5%) | 109(49.8%) |  |  |
|  | CC | 145 (19.5%) | 54 (18.6%) | 1.03 (0.83-1.27) | 0.781 |  | 191(22.0%) | 41(18.7%) | 0.93 （0.73-1.18） | 0.542 |
|  |  |  |  |  |  |  |  |  |  |  |
| rs3115759 | AA | 359 (47.5%) | 133 (45.7%) |  |  |  | 409(47.1%) | 110(50.2%) |  |  |
|  | AG | 318 (42.1%) | 130 (44.7%) |  |  |  | 375(43.2%) | 90(41.1%) |  |  |
|  | GG | 78 (10.4%) | 28 (9.6%) | 0.99 (0.79-1.23) | 0.901 |  | 85(9.7%) | 19(8.7%) | 0.82 （0.63-1.06） | 0.127 |

NT, normotensive subjects. HT, hypertensive subjects.

^*^*P* values were adjusted for age, body mass index and fasting plasma glucose.

**Supplemental Table 4 Analysis for the association of haplotypes with hypertension in non-diabetic subjects stratified by gender**

|  | Haplotype | Men | | | | |  | Women | | | | |
| --- | --- | --- | --- | --- | --- | --- | --- | --- | --- | --- | --- | --- |
|  |  | Frequency | | OR (95% CI) | *P* | *P^*^* |  | Frequency | | OR (95% CI) | *P* | *P^*^* |
|  |  | HT | NT |  |  |  |  | HT | NT |  |  |  |
| Stage 1 | CCA | 0.48 | 0.38 | 1.47(1.09-2.00) | 0.012 | 0.032 |  | 0.43 | 0.44 | 0.98(0.81-1.19) | 0.853 | 0.790 |
|  | CTG | 0.30 | 0.36 | 0.77(0.55-1.06) | 0.106 | 0.080 |  | 0.32 | 0.31 | 1.03(0.84-1.27) | 0.756 | 0.994 |
|  | TTA | 0.16 | 0.20 | 0.75(0.50-1.12) | 0.159 | 0.381 |  | 0.19 | 0.19 | 1.03(0.80-1.31) | 0.839 | 0.920 |
|  | CTA | 0.07 | 0.06 | 1.06(0.57-1.97) | 0.847 | 0.624 |  | 0.06 | 0.06 | 0.91(0.61-1.36) | 0.642 | 0.817 |
|  |  |  |  |  |  |  |  |  |  |  |  |  |
| Stage 2 | CCA | 0.45 | 0.47 | 0.97 (0.81-1.15) | 0.709 | 0.725 |  | 0.44 | 0.46 | 0.96 (0.85-1.08) | 0.486 | 0.129 |
|  | CTG | 0.34 | 0.32 | 1.05 (0.83-1.32) | 0.684 | 0.734 |  | 0.29 | 0.31 | 0.93 (0.79-1.09) | 0.379 | 0.608 |
|  | TTA | 0.17 | 0.16 | 1.11 (0.77-1.59) | 0.577 | 0.674 |  | 0.22 | 0.19 | 1.16 (0.95-1.42) | 0.149 | 0.053 |
|  | CTA | 0.04 | 0.06 | 0.71 (0.34-1.48) | 0.348 | 0.495 |  | 0.06 | 0.05 | 1.19 (0.76-1.85) | 0.446 | 0.391 |

Haplotypes were constructed in the order of rs2235307, rs2235306 and rs3115759.

NT, normotensive subjects. HT, hypertensive subjects.

^*^*P* values were adjusted for age, body mass index and fasting plasma glucose.

**Supplemental Table 5 Meta-analysis for the effect of three SNPs on hypertension in non-diabetic subjects**

|  | Men | | |  | Women | | |
| --- | --- | --- | --- | --- | --- | --- | --- |
|  | rs2235307 | rs2235306 | rs3115759 |  | rs2235307 | rs2235306 | rs3115759 |
| Stage 1 | 0.90 (0.73-1.12) | 1.19 (1.01-1.40) | 0.86 (0.72-1.02) |  | 0.98 (0.75-1.27) | 1.03 (0.83-1.27) | 0.99 (0.79-1.23) |
| Stage 2 | 1.05 (0.82-1.35) | 0.97 (0.81-1.16) | 1.03 (0.85-1.25) |  | 1.35 (1.02-1.80) | 0.93 (0.73-1.18) | 0.82 (0.63-1.06) |
| Overall | 0.97 (0.82-1.14) | 1.08 (0.96-1.22) | 0.93 (0.82-1.06) |  | 1.13 (0.94-1.37) | 0.98 (0.84-1.15) | 0.91 (0.77-1.08) |
| Test for heterogeneity | *I*^2^=0% (*P*=0.35) | *I*^2^=63% (*P*=0.10) | *I*^2^=51% (*P*=0.15) |  | *I*^2^=62% (*P*=0.10) | *I*^2^=0% (*P*=0.52) | *I*^2^=13% (*P*=0.28) |
| Test for overall effect | *P*=0.67 | *P*=0.19 | *P*=0.27 |  | *P*=0.20 | *P*=0.84 | *P*=0.27 |

Genetic effects are presented as odds ratio (95% confidence interval).

**Supplemental Table 6 The statistical power to of the current study to detect a given OR**

| SNP | OR | Statistical power | | | | |
| --- | --- | --- | --- | --- | --- | --- |
|  |  | Diabetic group | |  | Non-diabetic group | |
|  |  | Men | Women |  | Men | Women |
| rs2235307 | 1.1 | 0.16 | 0.13 |  | 0.14 | 0.16 |
|  | 1.2 | 0.47 | 0.37 |  | 0.38 | 0.45 |
|  | 1.3 | 0.78 | 0.64 |  | 0.67 | 0.76 |
|  | 1.4 | 0.94 | 0.84 |  | 0.87 | 0.93 |
|  |  |  |  |  |  |  |
| rs3115759 | 1.1 | 0.25 | 0.21 |  | 0.21 | 0.25 |
|  | 1.2 | 0.69 | 0.59 |  | 0.59 | 0.68 |
|  | 1.3 | 0.95 | 0.88 |  | 0.87 | 0.94 |
|  | 1.4 | 0.99 | 0.98 |  | 0.98 | 0.99 |
|  |  |  |  |  |  |  |
| rs2235306 | 1.1 | 0.27 | 0.22 |  | 0.22 | 0.26 |
|  | 1.2 | 0.72 | 0.63 |  | 0.63 | 0.71 |
|  | 1.3 | 0.96 | 0.91 |  | 0.91 | 0.95 |
|  | 1.4 | 0.99 | 0.99 |  | 0.98 | 0.99 |
